# Monkeypox exposure in patients living with HIV (PLWH): Epidemiological and clinical significance and a summary of response recommendations

**DOI:** 10.1016/j.nmni.2022.101057

**Published:** 2022-12-06

**Authors:** Farbod Semnani, Amirmasoud Rayati Damavandi, Seyed Sahab Aarabi

**Affiliations:** Students' Scientific Research Center, Tehran University of Medical Sciences, Tehran, Iran; School of Public Health, Tehran University of Medical Sciences, Tehran, Iran

## Abstract

• Immunosuppression and human immunodeficiency virus (HIV) infection may be associated with a more severe form of Monkeypox.
• The suspected sexual transmission of Monkeypox may change our perception of the Monkeypox typical manifestations.
• We summarized the recommendations for PLWH with Monkeypox exposure to provide a coherent source for the clinical approach.

The recent monkeypox outbreak in non-endemic areas, a potentially global epidemic raised by the World Health Organization (WHO) on May 21, 2022, has inflicted nearly 80 thousand patients as of November 10, 2022.

Although it has not been considered a sexually transmitted disease and almost anyone can contract the disease through mostly close contact, homosexuals, bisexuals, and MSM comprise most of the monkeypox cases. On the other hand, the Human immunodeficiency virus (HIV) is a common co-infection among this group. Hence, there is a need for public health policymakers to consider these risk factors and engage in risk communication [1].

In previous outbreaks, children, pregnant women, and immunocompromised individuals such as PLWH with poorly controlled diseases are at higher risks of disease progression and more severe outcomes. Nevertheless, more recent studies on emerging monkeypox cases among HIV-positive cases under effective ART treatment in Europe do not report a higher rate of hospitalization and mortality compared to the cases without HIV infection, suggesting the role of HIV-induced immunosuppression on patients' prognosis.

The epidemiologic shift in the recent outbreak in non-endemic countries might imply a paradigm shift in our perception of typical and atypical monkeypox presentations. The emergence of herald skin lesions in anogenital areas without the prodromal phase may strongly suggest sexual transmission via local inoculation of skin-to-skin contact and may not be associated with HIV infection. Hence, the monkeypox case definition and transmission route may need reevaluation as the outbreak continues.

## Summary of clinical recommendations

**Fig. 1:**
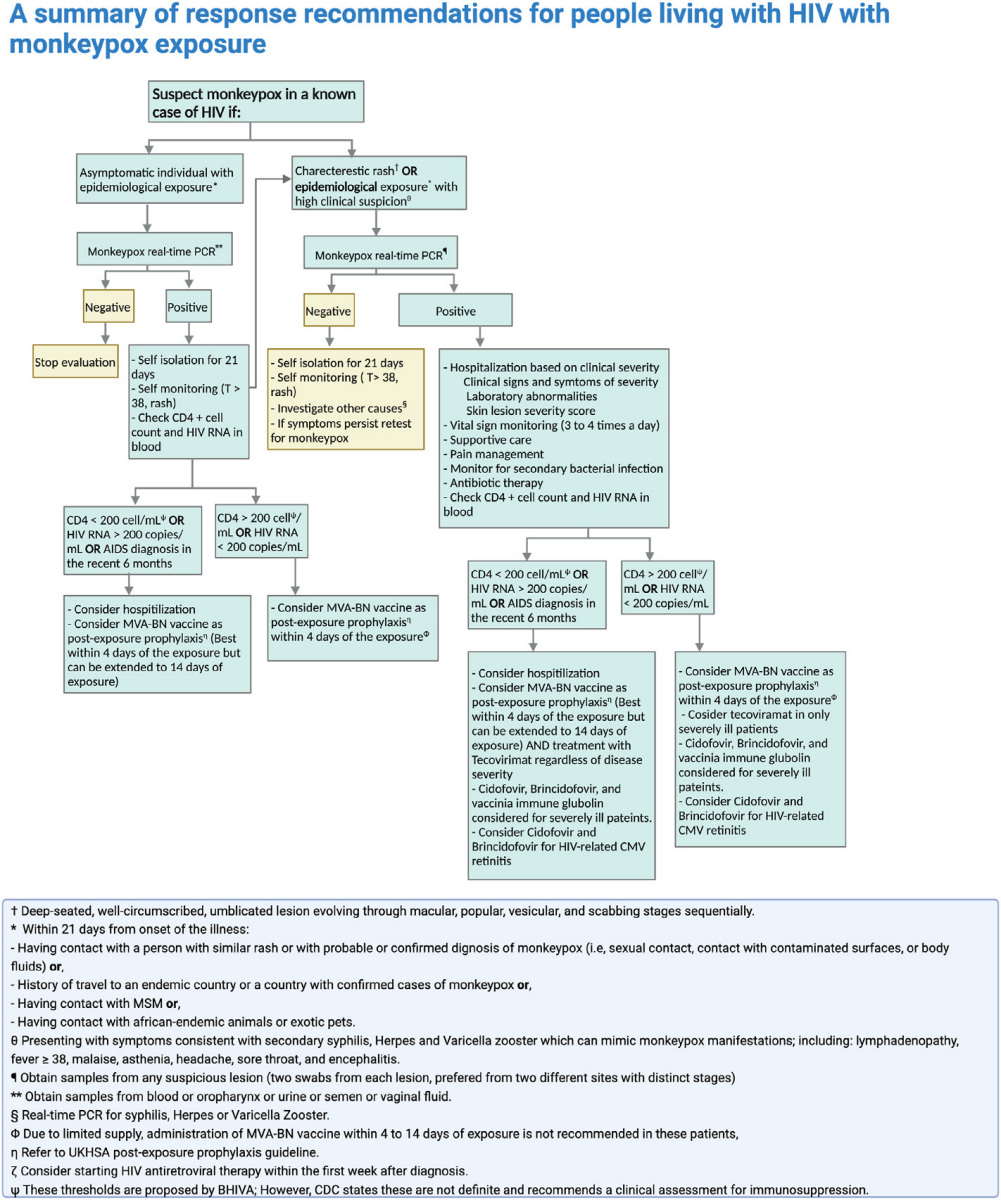
A summary of response recommendations for people living with HIV with monkeypox exposure, MSM = men having sex with men, MVA-BN= Modified Vaccinia Ankara - Bavarian Nordic.

Herein, we propose a short summary of the recommendations released for monkeypox management, i.e. CDC, The British HIV Association (BHIVA), and the World Health Organization (WHO), with a focus on PLWH with suspected monkeypox exposure (Fig. 1). If a new diagnosis of HIV infection is established, ART should be started as soon as possible and best within the first week [2]. Should the diagnosis of monkeypox and HIV be made simultaneously, these patients must be prioritized for the most urgent treatments [2]. The clinicians should suspect infection with monkeypox in a person living with HIV with a characteristic rash, even without apparent epidemiological reference. Diagnosis is confirmed by nucleic acid amplification testing (conventional or real-time PCR) taken from any suspicious skin or mucosal lesion. After the diagnosis is confirmed, 21-day isolation is suggested for all patients [2]. However, as immunocompromised PLWH with poorly controlled illness (CD4 < 200 cell/mL or HIV RNA >200 copies/mL) or individuals with AIDS diagnosis in the past six months may have prolonged virus shedding from the upper respiratory tract, a further clinical assessment is required regarding the termination of the isolation [2,3]. Nonetheless, due to data scarcity, strict thresholds for CD4 count or HIV RNA copies should not be considered alone and the overall risk for immunosuppression should be clinically evaluated [3,4]. Moreover, after the monkeypox diagnosis is established, clinicians should consider the hospitalization of these groups for closer monitoring, even if they remain asymptomatic [2].

Post-exposure vaccination with ACAM2000, a live replication-competent FDA-approved vaccine for monkeypox, is recommended against for PLWH due to adverse events like progressive vaccinia. MVA-BN (JYNNEOS), a third-generation replication-deficient smallpox vaccine recently authorized in the USA, Canada, and the EU, should be prioritized for these patients. Regarding treatment, tecovirimat, vaccinia immune globulin (VIG), cidofovir, and brincidofovir can be considered for HIV + patients who are severely ill [5]. Tecovirimat, an inhibitor of the viral envelope protein VP37, may be considered the first-line option for treating symptomatic immunosuppressed HIV + monkeypox patients regardless of disease severity [5]. Tecovirimat is approved by the European Medicines Agency for monkeypox and in the USA, Canada, and Europe for human smallpox disease. Nonetheless, it is still not FDA-approved for monkeypox treatment and may be administered through non-research expanded access Investigational New Drug (EA-IND) protocol. Although proven safe, its efficacy has not been evaluated in human trials. Tecovirimat and brincidofovir have potential drug-drug interactions with antiretrovirals and should be administered cautiously. However, none of these considerations should hinder the co-administration of ART and monkeypox-specific treatments [4].

The evidence is still patchy regarding monkeypox in possibly high-risk groups, including PLWH, without receiving appropriate ART or immunocompromised. Therefore, the figure provided may help clinicians and policymakers to benefit from a more coherent illustration of the current recommendations proposed by different sources.

## Ethics approval and consent to participate

Not applicable.

## Consent for publication

A signed license to publish is provided.

## Availability of data and materials

Not applicable.

## Funding

This study has received no funding.

All authors participated in preparing the final draft of the manuscript, revised the manuscript, and critically assessed the academic content. All authors have read and approved the manuscript and confirmed the accuracy or integrity of any part of the work.

## CRediT authorship contribution statement

**Farbod Semnani:** Conceptualization, Data curation, Investigation, Methodology, Supervision, Validation, Writing – original draft, Writing – review & editing, All authors participated in preparing the final draft of the manuscript, revised the manuscript, and critically assessed the academic content. All authors have read and approved the manuscript and confirmed the accuracy or integrity of any part of the work. **Amirmasoud Rayati Damavandi:** Conceptualization, Data curation, Investigation, Methodology, Validation, Writing – original draft, Visualization, All authors participated in preparing the final draft of the manuscript, revised the manuscript, and critically assessed the academic content. All authors have read and approved the manuscript and confirmed the accuracy or integrity of any part of the work. **Seyed Sahab Aarabi:** Conceptualization, Data curation, Investigation, Resources, Methodology, Validation, Writing – original draft, All authors participated in preparing the final draft of the manuscript, revised the manuscript, and critically assessed the academic content. All authors have read and approved the manuscript and confirmed the accuracy or integrity of any part of the work.

## Declaration of competing interest

The authors do not declare a conflict of interests.
